# BSA coated gold nanoparticles exhibit size dependent interaction with lung cancer (A549) cells

**DOI:** 10.1186/1755-8166-7-S1-P83

**Published:** 2014-01-21

**Authors:** Rahul Purohit, NV Srikanth Vallabani, Ritesh K Shukla, Ashutosh Kumar, Alok Dhawan, Sanjay Singh

**Affiliations:** 1 Institute of Life Sciences, School of Science and Technology, Ahmedabad University, Ahmedabad – 380009, Gujarat, India

## Background

Due to high surface to volume ratio and other unique properties, nanomaterials interact within living system (cells, tissues, organisms) significantly different. Therefore, developing a rational basis for investigation and understanding about how these nanomaterials interact with living system is still a fundamental challenge. Further, it has been well documented that nanomaterials get coated by the proteins present in their surrounding thus constructing a “protein corona”. This event also plays a major role in determining the interaction of nanomaterials with cells/tissues. Therefore, to probe this we have chosen BSA coated gold nanoparticles (AuNPs) as a model system to study the interaction with a mammalian lung cell culture model system.

## Materials and methods

AuNPs of different sizes (15, 30, 50 and 70 nm) were synthesized using sodium citrate as a reducing agent. Size distribution, zeta potential and stability of coated (AuNPSBSA) as well as uncoated AuNPs were determined by dynamic light scattering (DLS) and UV-vis spectroscopy. Cytotoxicity response was assessed by neutral red uptake (NRU) and MTT [3-(4, 5-dimethylthiazoyl-2-yl)-2, 5-diphenyltetrazolium bromide] assays.

## Results

Zeta potential values for AuNPs of different sizes were found negative, which was decreased significantly after BSA coating. Further, the stability of AuNPSBSA in saline solution (1M and 500 mM NaCl) and cell culture media (DMEM-F12), which showed that BSA coated AuNPs were stable in both saline solution and serum containing culture media, as compared to uncoated AuNPs. Cytotoxicity studies revealed that BSA coated and bare AuNPs were non-toxic up to 50 µM, however, at higher concentration (above 50 µM) bare AuNPs were found to be more toxic as compared to AuNPSBSA. Further, the cytotoxicity of AuNPs on A549 cells was also found to be size dependent.

## Conclusion

This study demonstrates the size and BSA coating on AuNPs significantly control the cytotoxicity on lung cancer cells. BSA coating imparts the biocompatibility thus non toxic nature of these particles even at higher concentration. However, the relation between cytotoxicity and internalization of AuNPs (bare and BSA coated) in A549 cells remains to be seen.

